# Bone marrow vs Wharton’s jelly mesenchymal stem cells in experimental sepsis: a comparative study

**DOI:** 10.1186/s13287-019-1295-9

**Published:** 2019-06-27

**Authors:** Caroline Laroye, Amir Boufenzer, Lucie Jolly, Lisiane Cunat, Corentine Alauzet, Jean-Louis Merlin, Clémence Yguel, Danièle Bensoussan, Loïc Reppel, Sébastien Gibot

**Affiliations:** 1 CHRU de Nancy, Unité de Thérapie Cellulaire et banque de Tissus, Allée du Morvan, 54500 Vandoeuvre-lès-Nancy, France; 2 INSERM UMRS-1116, Vandoeuvre-lès-Nancy, France; 3 CNRS, UMR 7365, 54500 Vandoeuvre-lès-Nancy, France; 4 Université de Lorraine, 54000 Nancy, France; 5 INOTREM, 54500 Vandoeuvre-lès-Nancy, France; 6 EA 7300 Stress Immunité Pathogènes, 54500 vandoeuvre-lès-Nancy, France; 7 Service de Biopathologie - Unité de Biologie des Tumeurs, Institut de Cancérologie de Lorraine, 54500 Vandœuvre-lès-Nancy, France; 8 CHRU de Nancy, laboratoire anatomie et cytologie pathologiques, 54500 Vandoeuvre-lès-Nancy, France; 9 CHRU de Nancy, Service de Réanimation Médicale, Hôpital Central, 54000 Nancy, France

**Keywords:** Mesenchymal stem cells, Tissue source, Wharton’s jelly, Bone marrow, Sepsis

## Abstract

**Background:**

The use of mesenchymal stem cells (MSCs) is being extensively studied in clinical trials in the setting of various diseases including diabetes, stroke, and progressive multiple sclerosis. The unique immunomodulatory properties of MSCs also point them as a possible therapeutic tool during sepsis and septic shock, a devastating syndrome associated with 30–35% mortality. However, MSCs are not equal regarding their activity, depending on their tissue origin. Here, we aimed at comparing the in vivo properties of MSCs according to their tissue source (bone marrow (BM) versus Wharton’s jelly (WJ)) in a murine cecal ligation and puncture (CLP) model of sepsis that mimics a human peritonitis. We hypothesized that MSC properties may vary depending on their tissue source in the setting of sepsis.

**Methods:**

CLP, adult, male, C57BL/6 mice were randomized in 3 groups receiving respectively 0.25 × 10^6^ BM-MSCs, 0.25 × 10^6^ WJ-MSCs, or 150 μL phosphate-buffered saline (PBS) intravenously 24 h after the CLP procedure.

**Results:**

We observed that both types of MSCs regulated leukocyte trafficking and reduced organ dysfunction, while only WJ-MSCs were able to improve bacterial clearance and survival.

**Conclusion:**

This study highlights the importance to determine the most appropriate source of MSCs for a given therapeutic indication and suggests a better profile for WJ-MSCs during sepsis.

## Background

Mesenchymal stem cell (MSC) administration is being extensively studied in clinical trials in the setting of many different disorders such as graft versus host disease, cardiomyopathy, diabetes, stroke, bronchopulmonary dysplasia, progressive multiple sclerosis, or osteoarthritis. Indeed, MSCs are an attractive therapeutic candidate for several reasons: these cells display immunomodulatory, anti-inflammatory, antibacterial, and differentiation properties [1]. Their isolation and expansion are both easy and fast as compare to other stem cells like embryonic stem cells. They are devoid of MHC class II antigens and express only low levels of MHC class I antigens, allowing their use in an allogeneic setting due to their low immunogenicity [2]. Finally, several clinical trials reported no adverse events after MSC infusion, describing those cells as safe for clinical use [3].

Since their discovery in the bone marrow (BM) by Friedenstein’s team in 1976, MSCs have been found in the skeletal muscle, adipose tissue [4], dental pulp, trabecular bone synovial membrane, lungs [5], heart [6], synovial membrane, trabecular bone, periosteum [7, 8], and menstrual blood [9], as well as in different birth tissues, including the amniotic fluid and membrane [10], placenta [11], umbilical cord blood [12], and Wharton’s jelly (WJ) [13].

The International Society for Cellular Therapies defined MSCs as (i) CD34^neg^ CD45^neg^ HLADR^neg^ CD90^+^ CD73^+^ CD105^+^ cells with (ii) plastic adherence (iii) and ability to differentiate into osteocytes, adipocytes, and chondrocytes. However, despite this consensual definition, MSCs remain a very heterogeneous cell population and large variations in their properties, partly related to their tissue source, have been described [14]. For example, Alcayaga et al. demonstrated a superior frequency of menstrual stem cell fibroblast colony-forming units as compared to bone marrow stem cells (BM-MSCs) [15]. Paneppucci et al. described better osteogenic differentiation when MSCs were derived from BM as compared to WJ [16]. Li et al. found that MSCs from birth tissues have stronger immunomodulatory properties than BM-derived cells [17]. Accordingly, it is essential to determine the most suitable source of MSCs to get the best-expected effect depending on the therapeutic indication considered.

Sepsis, defined as life-threatening organ dysfunction caused by a deregulated host response to infection, is a leading cause of admission to intensive care units and is associated with high mortality rates [18, 19]. Unfortunately, due to its complex physiopathology, there is still no specific treatment for this syndrome. Mei et al. [20] were the first to suggest that MSCs improve survival and decrease organ failure in a mouse model of endotoxemia, and subsequent studies showed that MSCs can increase bacterial clearance [21], modulate cytokine production [22–25], and improve renal, pulmonary, liver, cardiac, and muscular functions [21, 26–29]. Although promising, these studies used MSCs derived from adult tissues (BM and adipose tissue) exhibiting many drawbacks with regard to their potential for clinical applications: the number of adult MSC donors is limited, and adult MSCs remain difficult to produce. By contrast, fetal tissues, and particularly the umbilical cord, are much easier to obtain and MSCs are present in large numbers in these tissues and can be expanded [30].

Therefore, in this study, we compared the in vivo properties of MSCs according to their tissue source: BM versus WJ, which is an attractive source due to its abundance, during a cecal ligation and puncture model of sepsis that mimics a human peritonitis with immune deregulation, organ injury bacterial invasion [31]. We hypothesized that MSC properties may vary depending on their tissue source in the setting of sepsis.

## Methods

### MSC preparation

Umbilical cords were collected at Nancy Maternity Hospital from new mothers who had signed an informed consent form in compliance with the French national legislation regarding human sample collection, manipulation, and personal data protection. Umbilical cord removal was performed in parallel in a context of hematopoietic stem cell allograft. Briefly, after the removal of umbilical cord vessels, WJ was cut into small pieces and plated in a six-well plate with complete medium (minimal essential medium alpha (αMEM; Lonza, Walkersville, MD, USA) supplemented with 10% fetal bovine serum (FBS), 2 mM glutamine, 100 IU/mL penicillin, 100 μg/mL streptomycin, and 2.5 μg/mL amphotericin B). After 7 days, pieces were removed and culture continued until passage 3.

Bone marrow-MSCs were isolated from a sample of healthy human BM collected in a hematopoietic stem cell allograft context, after donors and patients’ informed consent in compliance with national legislation regarding human sample collection, manipulation, and personal data protection. Nuclear cells were seeded at 50000/cm^2^ in a complete medium. After 2 days, cultures were washed to eliminate non-adherent cells, the medium replaced, and cultures continued until passage 1.

MSC cultures were carried out at 37 °C in hypoxic conditions (5% of O_2_ and 5% of CO_2_).

All donors met the criteria for HSC allogeneic transplant: for example, no medical history against donation, negative serology for less than 1 month, and age lower than 50 years. To minimize the impact of donor variabilities, we used 6 donors from each MSC source.

At the end of culture, MSCs were washed with HBSS (Hanks balanced salt solution) and detached by trypsinization. One million MSCs were labeled with anti-CD90, CD73, CD44, CD105, CD34, CD45, CD11b, CD19, and HLA-DR mAbs (Stemflow hMSC Analysis kit, Becton Dickinson, Franklin Lakes, USA) for characterization; the remaining cells were frozen and stored in vapor phase nitrogen. For their use in our experimental sepsis model, MSCs were administered immediately after thawing to reflect the clinical setting.

### Functional characterization of MSC

As a control of MSC characteristics, osteogenic differentiation was induced by seeding MSCs at a density of 3100 cells/cm^2^ and by culturing them for 28 days in an osteogenic induction medium (Lonza, USA). After 28 days, the samples were fixed in 4% paraformaldehyde and then embedded in paraffin before being stained with alizarin red. To induce adipocyte differentiation, 21,000 MSCs/cm^2^ were seeded onto a 24-well plate. When 100% confluence was reached, 3 cycles of induction/maintenance were performed. One cycle of induction/maintenance consisted in 3 day-culture in induction medium (Lonza, USA), followed by 1 to 3 days of culture in maintenance medium (Lonza). After 3 cycles of induction/maintenance, the cells were cultured for 7 days in complete maintenance medium (Lonza, USA) before being stained with oil red.

### Cecal ligation and puncture polymicrobial sepsis model

Experiments were performed in compliance with the National Institute of Health guidelines on the use of laboratory animals and evaluated by our Institutional Animal Care and Use Committee (CELMEA-CE2A-66). Cecal ligation and puncture (CLP) was performed, as previously described [32]. Eight- to 10-week-old male C57BL/6 mice were anesthetized by isoflurane inhalation (4% isoflurane for induction; 1.5% isoflurane for maintenance). After laparotomy, the distal end of the cecum was ligated, a single perforation was performed with an 18-gauge needle, and a small amount of stool was taken out. The cecum was then replaced into the peritoneal cavity, and the abdominal incision was sutured in two layers with 4.0 nylon suture. Five hundred microliters of 0.9% NaCl was administered sub-cutaneously for fluid resuscitation. Mice were then immediately randomized in three groups: BM-MSC, WJ-MSC, and phosphate-buffered saline (PBS) by a person who did not perform surgery. Twenty-four hours after CLP procedure, 2.5 × 10^5^ MSCs in 150 μl of PBS or 150 μl PBS alone were slowly administered into the retro-orbital sinus under sevoflurane anesthesia.

### Inflammation studies

Forty-eight hours or 7 days after CLP procedure, animals were sacrificed by pentobarbital i.p. injection and blood and organs were harvested. Blood count was determined by a hemocytometer, and plasma concentrations of IL1β, IL-6, IL-10, IFNγ, and TNFα were measured by multiplex immunoassays (Bio-Plex Pro Mouse Th1 cytokine, Biorad, France) according to the manufacturer’s recommendations. The same protocol was carried out on healthy mice (H0).

Leukocyte trafficking was analyzed by flow cytometry as previously described [33]. The spleen and liver were crushed in HBSS and filtered on a 70-μm nylon filter. The bone marrow was extracted from the femur, after the bone has been clipped, by rapidly injecting 1 ml of PBS into the medullary cavity. The lungs were cut into fine pieces and incubated in a cocktail of collagenase I and DNase I at 37 °C for 45 min before being crushed and filtered. After washing, a cell count was performed by a hemocytometer with Trypan blue staining (BioRad). Cell suspensions were labeled with a combination of anti-CD4-PerCP, CD25-PE, CD11b-Vioblue, Ly6C-FITC, Ly6G-PE, FoxP3-APC, and CD45-PerCP mAbs (Miltenyi, France) after permeabilization according to the manufacturer’s recommendations. Data were acquired on a Gallios FACS analyzer (Beckman Coulter). The same protocol was carried out on healthy mice (H0).

### Bacterial count

The blood and spleen were obtained 48 h after the CLP procedure. The blood and crushed spleen were plated in serial log dilutions on blood agar plates. After plating, tryptic soy agar plates were incubated at 37 °C aerobically for 24 h and anaerobically for 48 h and colony-forming units (CFUs) were counted. Results are expressed as CFUs per milliliter of blood or per gram of spleen.

### Analysis of organ injuries

Organ dysfunction was determined by measurement of biochemical indicators of organ functions in plasma samples and by histology scores. Plasma concentrations of urea and creatinine (indicators of renal dysfunction), alanine aminotransferase (ALT) and alkaline phosphatase (ALKP) (indicators of liver dysfunction), and amylase and glycemia (indicators of pancreatic dysfunction) were analyzed by an automate (VetTest GHP, Idexx, Saint-Denis, France) after the animals’ sacrifice 2 or 7 days after the CLP procedure.

Forty-eight hours after CLP, the lungs, liver, kidneys, and spleen were fixed in 4% paraformaldehyde during 24 h at 4 °C, dehydrated through a series of ethanol concentrations, cleared with toluene, embedded in paraffin wax, and cut into 5-mm-thick sections with a Leica micro-tome (RM2135, Leica, France). For histological examination, each specimen was stained with hematoxylin-erythrosin-saffron, mounted on glass slides, and visualized on an optical microscope (DMD 108, Leica, France).

Histology scores were performed by an experienced pathologist blinded to the treatment administered. The scoring system was adapted from [34, 35] and ranged from 0 to 9 for the lungs, 0 to 5 for the kidneys, 0 to 4 for the liver, and 0 to 3 for the spleen.

### Survival study

The same previously described CLP procedure was performed for the survival study. All mice received every 12 h 50 μg/g of imipenem (Braun, France) sub-cutaneously for the survival study in accordance with Alcayaga-Miranda’s work which described a cumulative effect of MSCs and antibiotics [15]. Animals were followed up to 7 days.

### Statistics

The normal distributions of the data were tested (Kolmogorov-Smirnov test), and data are presented as means ± SD. Between-group differences were tested for significance by two-way ANOVA with Bonferroni correction or Kruskal-Wallis test when appropriate. Analyses were performed using GraphPad Prism software.

## Results

All the MSCs used in this study presented the typical MSC phenotype CD14^neg^-CD34^neg^-HLA-DR^neg^-CD11b^neg^-CD19^neg^-CD73^+^-CD90^+^-CD105^+^-CD44^+^, differentiation capacities into osteocytes and adipocytes, and an adherence to plastic in accordance with the standards described by the International Society for Cellular Therapy.

### MSCs impact leukocyte trafficking

**Fig. 1:**
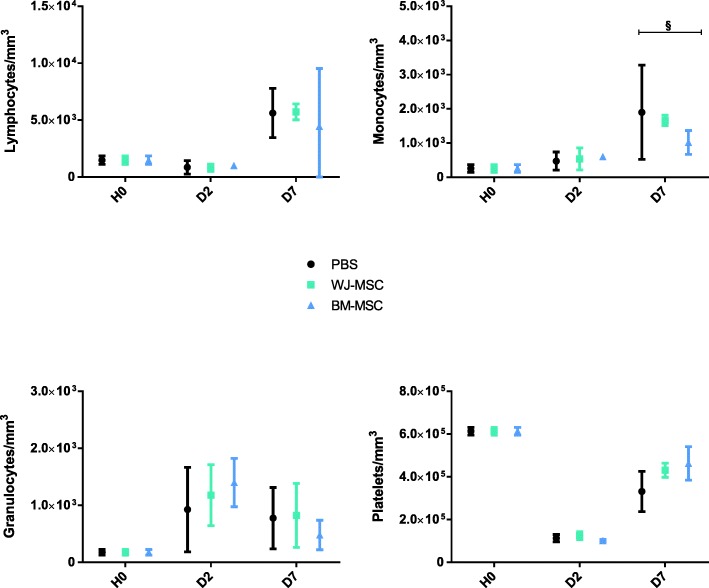
Evolution of blood cell count. Blood cell analysis was performed before (H0) and 2 and 7 days after the induction of sepsis. Results are expressed as mean ± SD (*n* = 3–6 per group). Group comparisons were analyzed by two-way ANOVA with Bonferroni correction. ^§^*p* < 0.05 BM-MSC versus PBS

Sepsis induced an early neutrophilia and a progressive monocytosis and lymphocytosis (Fig. 1). MSCs showed no effect on these disturbances, except for a reduced late (day 7) monocytosis with BM-MSCs. However, no significant difference was found between WJ-MSC and BM-MSC. Of note, sepsis was also associated with deep and rapid thrombocytopenia, on which MSCs had no effect.

**Fig. 2:**
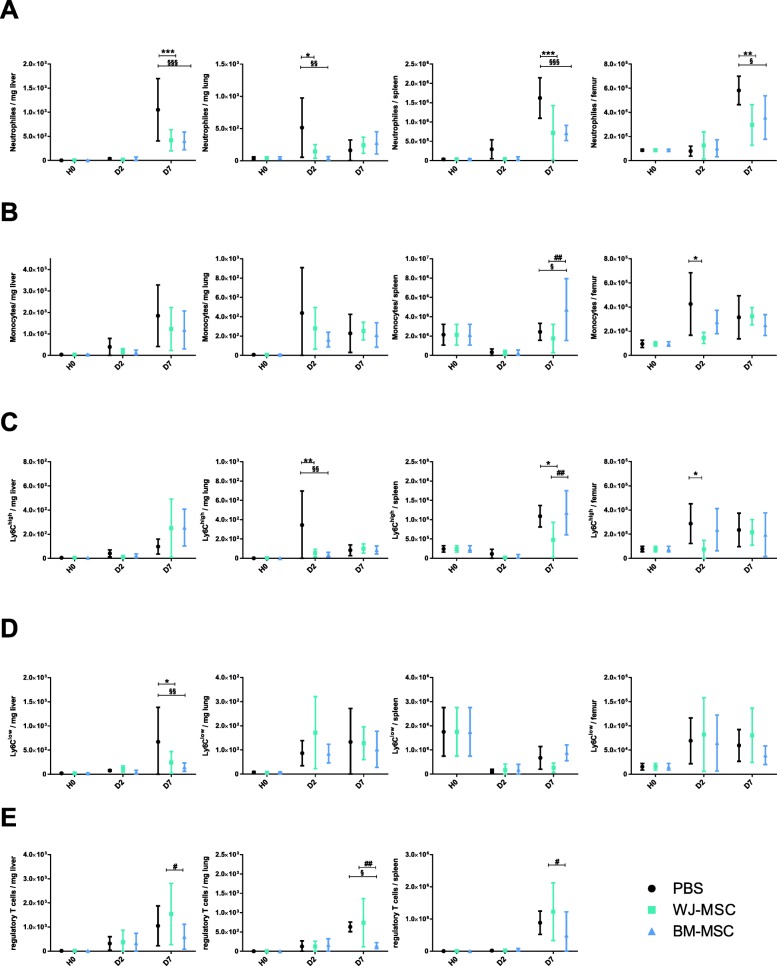
MSCs regulate leukocyte trafficking. Flow cytometric quantification of neutrophils (**a**), monocytes (**b**), monocytes Ly6C^high^ (**c**), monocytes Ly6C^low^ (**c**), and T regulatory lymphocytes (**e**) in the spleen, lung, liver, and bone marrow at different time points. Results are expressed as mean ± SD (*n* = 4–6 per group). *p < 0.05 WJ-MSC versus PBS; **p < 0.01 WJ-MSC versus PBS; ***p < 0.001 WJ-MSC versus PBS; ^§^*p* < 0.05 BM-MSC versus PBS; ^§§^*p* < 0.01 BM-MSC versus PBS; ^§§§^*p* < 0.001 BM-MSC versus PBS; ^#^*p* < 0.05 BM-MSC versus WJ-MSC; ^##^*p* < 0.01 BM-MSC versus WJ-MSC

We next investigated leukocyte trafficking in various organs (Fig. 2). Following CLP, neutrophils progressively populated the liver, spleen, lungs, and BM. This was partially prevented by MSCs regardless of their origin (Fig. 2a). Monocytes were also recruited by the lungs and the liver, though to a less extent in MSC-treated animals (Fig. 2b). In mouse, monocytes are classically categorized as “pro-inflammatory” Ly6C^high^ and “anti-inflammatory” Ly6C^low^. Mobilization of Ly6C^high^ monocytes to the lung was reduced in mice receiving MSCs regardless of their origin while Ly6C^low^ was largely unaffected except in the liver (Fig. 2c, d). Likewise, we observed a significant reduction of Ly6C^high^ monocytes in BM in the WJ-MSC group in comparison to the PBS group. However, no significant difference was found between WJ-MSC and BM-MSC. Regarding Treg-cells, known to favor resolution of inflammation, WJ-MSC-treated mice displayed higher numbers in their lungs, liver, and BM, than BM-MSC-treated mice though with no significant differences as compared to control animals (Fig. 2e). Altogether, these data show that MSCs are able to impact leukocyte trafficking, towards a more “anti-inflammatory” profile conferred after treatment by WJ-derived cells.

### MSCs do not dampen systemic inflammation but reduce organ injury

**Fig. 3:**
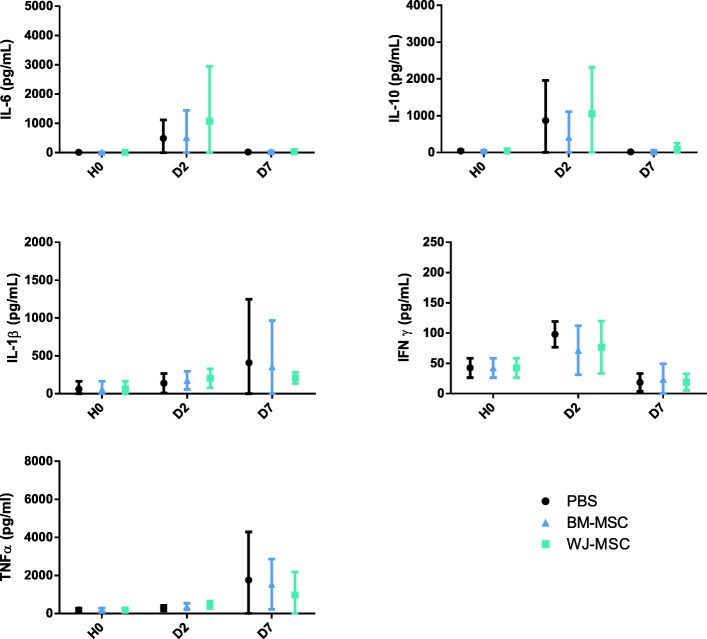
MSCs have no effect on plasma cytokine concentrations. Plasma concentrations of IL1β, IL-6, IL-10, IFNγ, and TNFα were determined by multiplex assay at baseline (H0) and 2 and 7 days after CLP. Results are expressed as mean ± SD(*n* = 5–10 per group). Group comparisons were analyzed by two-way ANOVA with Bonferroni correction

**Fig. 4:**
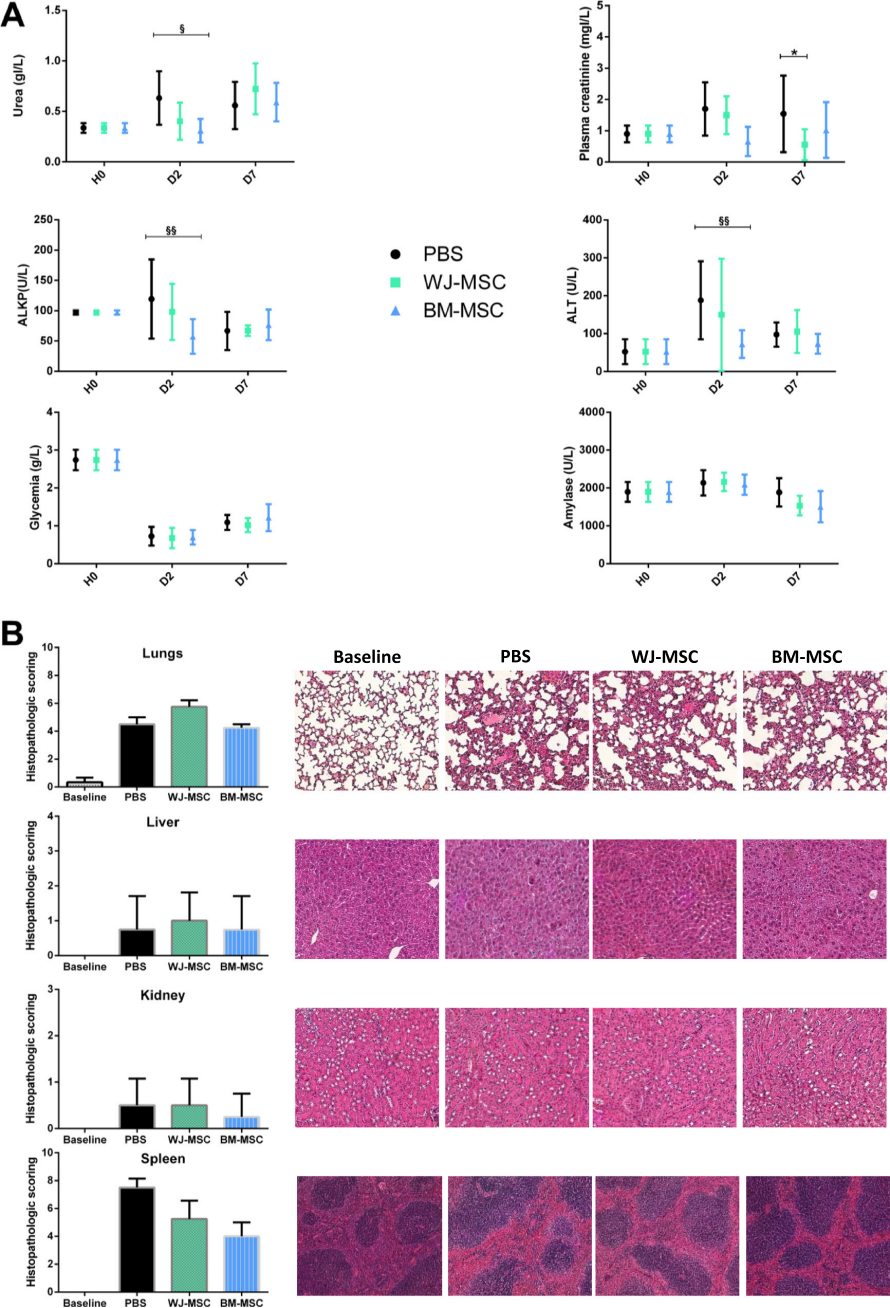
Effects of MSCs on organ dysfunction. Plasma concentrations of blood urea, creatinine, ALT, ALKP, amylase, and glycemia were measured at baseline and 2 or 7 days after CLP procedure (*n* = 3–7 per group) (**a**). The lungs, livers, kidneys, and spleens were harvested 48 h after CLP, and a pathologist blinded to the treatment group performed histology scoring (*n* = 4 per group) (**b**). Group comparisons were analyzed by two-way ANOVA with Bonferroni correction. *p < 0.05 WJ-MSC versus PBS; ^§^*p* < 0.05 BM-MSC versus PBS; ^§§^*p* < 0.01 BM-MSC versus PBS

As expected, sepsis induced an acute increase in both inflammatory (TNFα, IL1β, IL-6) and anti-inflammatory (IL-10) plasma cytokine concentrations (Fig. 3). Surprisingly, we observed no effect of MSCs on cytokine levels. Moderate kidney and liver dysfunctions develop after CLP (Fig. 4a). These early disturbances were partly prevented by MSCs regardless of their source. Histology studies of the lungs, spleens, kidneys, and livers revealed no significant differences between groups (Fig. 4b).

### MSCs improve bacterial clearance

**Fig. 5:**
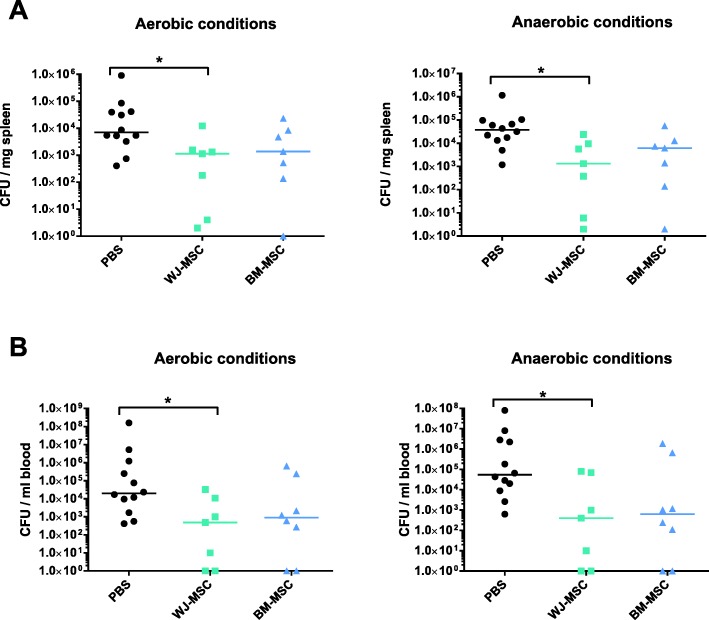
WJ-MSCs enhance bacterial clearance. Spleen (**a**) and blood (**b**) bacterial CFU were assessed 48 h after the induction of sepsis. Results are expressed as median (*n* = 7–12 per group). *p < 0.05 WJ-MSCs versus PBS. Group comparisons were analyzed by Kruskal-Wallis test

We observed an important reduction of blood and spleen bacterial load as compared to controls after the administration of WJ-MSCs (Fig. 5). Although the trend was similar for BM-MSCs, it did not reach significance. Of note, 100% of control animals were bacteremic compared to 71% and 75% in the WJ- and BM-MSC groups respectively (Fig. 5b).

### MSCs improve survival

**Fig. 6:**
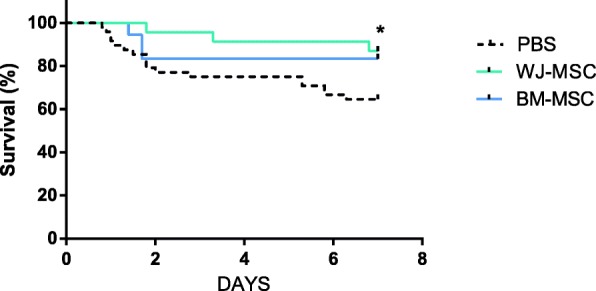
WJ-MSCs improve survival. Kaplan-Meier estimate of survival after CLP (*n* = 18–48 per group). Survival curves were compared using the log-rank test. Group comparisons were analyzed by Wilcoxon test *p < 0.05 WJ-MSCs versus PBS

Finally, we investigated the effects of MSCs on survival (Fig. 6). In the control group (antibiotics and fluid resuscitation), lethality reached 36% at day 7, but only 13% and 17% in the WJ- and BM-MSC-treated animals respectively. Significance was only obtained for the WJ-MSC group (Wilcoxon test, *p* = 0.04). No significant difference was found between WJ-MSC and BM-MSC groups.

## Discussion

Although the use of MSCs as a therapeutic tool has generated a great enthusiasm, important questions remain especially regarding their optimal tissue source that may depend on the target disorder. Sepsis is one of the diseases under scrutiny for MSC administration. This syndrome being a vital emergency, allogenic MSC banks will be necessary and the optimal MSC source must be determined. We aimed here to address whether in vivo MSC properties during sepsis could change according to their adult or fetal origin.

Indeed, we observed slightly different effects of MSCs depending on their tissue source. First, WJ-MSCs seem to be more potent than BM-MSCs to modulate leukocyte trafficking, conferring a more “anti-inflammatory” environment in organs with a lower neutrophil infiltration. Previous in vitro studies suggested that MSCs extracted from birth tissues have more immunomodulatory capacities than cells derived from adult tissues. Mareschi et al. studied the proliferative capacity of regulatory T cells during co-culture of mononuclear cells and MSCs from the birth tissue or bone marrow [36] and noted an increase in Treg cell proliferation with MSCs from birth tissue. Similarly, Barcia et al. observed that WJ-MSCs seemed to be less immunogenic and more immunosuppressive than BM-MSCs [37].

Although we demonstrated an important impact of MSCs on cellular traffic, no effects were noted on plasma cytokine concentrations. Our results contrast with previous findings demonstrating the MSC ability to increase anti-inflammatory cytokines and decrease pro-inflammatory cytokines [22–24, 38]. But it is crucial to underline the fact that we here administered MSCs as a treatment and not as a prophylactic agent: most if not all previous studies on MSCs and sepsis used cells at the same time (or even before) or just after the onset of sepsis. Here, we chose to give cells only 24 h after surgery (and thus analyzed cytokines at 48 h), to better mimic what could happen in the clinical setting. Therefore, our time window could have precluded from observing an MSC effect on cytokines, as their concentration peak at 24 h as observed in the Liu et al. study [39]. However, in a previous work in pigs, we also were unable to demonstrate an impact of an early WJ-MSC administration on plasma cytokines [29]. In a recent clinical trial investigating the effect of adipose tissue-derived MSCs after an intravenous injection of lipopolysaccharide (LPS) into healthy subjects, these cells did not decrease TNFα or IL6 release and only the higher MSC dose (4 × 10^6^/kg) increased plasma level of IL10 [40]. It is also important to note that in these studies MSCs have been cryopreserved. The impact of freezing is unclear [41–44], and therefore, an effect on MSC anti-inflammatory properties cannot be excluded.

The antibacterial properties of MSCs seem also affected by their origin. Indeed, even if no significant difference was found between WJ-MSC and BM-MSC, we observed a significant decrease in bacterial clearance only with WJ-MSCs in comparison to the PBS group. Differences in terms of antimicrobial peptide secretion have been previously described. For example, Alcayaga et al. showed that BM-MSCs secrete LL-37, hepcidin, and β defensins, whereas MSCs derived from the menstrual liquid secrete only hepcidin, and MSCs from the umbilical cord produce only β defensins [45]. Obviously, in vitro experiments cannot reflect the complex environment of sepsis and therefore the behavior of in vivo MSCs. Moreover, antibacterial properties of MSCs are not just the consequence of antimicrobial peptide production as they can also increase neutrophil and monocyte phagocytic index [46, 47], a mechanism complex to reproduce in vitro.

Although the better profile of MSCs regarding their immunomodulatory and antibacterial properties should have been translated into a dampening of organ dysfunction, this was oddly not observed. An explanation could stem from the fact that our model was not severe enough to drive severe organ failure, as suggested by modest biochemistry and histologic disorders in the sacrificed mice 2 days after the CLP procedure.

Finally, only WJ-MSCs were able to improve survival in comparison to the PBS group, though it is important to note that no difference was observed between WJ-MSC and BM-MSC groups.

In summary, we observed very slight differences between MSC capacities depending on tissue source such as their impact on regulatory T cells but none justifying the use of a source over the other in terms of potency. Currently, three phase 1 clinical trials have reported the safety of using MSCs during septic shock: two using MSCs derived from adult tissues (bone marrow and adipose tissue) and the third using MSCs from the umbilical cord [40, 48, 49]. These studies demonstrate that intravenous infusion of allogeneic MSCs in a septic context was safe. None adverse effect was observed even at high doses, regardless of their origin. However, to treat a syndrome with a high prevalence such as sepsis, the accessibility to the source of MSC is essential and the number of adult MSC donors is limited. In this context, WJ is a more advantageous tissue source of MSCs: donations of the umbilical cord are devoid of risk and abundant, the amount of MSCs in an umbilical cord is important, and their expansion is fast and quite easy.

This study presents some limitations. First, we used MSCs at different passages. BM-MSCs were cryopreserved at passage 1 while WJ-MSC at passage 3. Indeed, the BM-MSC senescence is earlier than the WJ-MSC senescence [50]. Although unlikely, whether this could have negatively impacted WJ-MSC properties is unknown. Second, we could have taken into account differences in terms of BM and WJ donor characteristics which have been found to influence MSC functions such as age, gender, weight, smoking status, diabetes, or obstetric factors [51–56]. Third, we tested a single dose of MSCs. However, even if the efficiency of higher amounts of cells is unknown, the dose of 0.25 × 10^6^ cells per animal corresponds to a high dose in humans (around 10 × 10^6^ cells/kg).

## Conclusion

We here showed that the effects of MSC administration in an in vivo model of murine sepsis marginally depend on their source, with an at least as potent profile conferred by WJ-MSCs.

## Data Availability

Data sharing is not applicable to this article as no datasets were generated or analyzed during the current study.

## Abbreviations

ALAT: Alanine aminotransferase
ALKP: Alkaline phosphatase
BM: Bone marrow
BM-MSCs: Mesenchymal stem cells derived from the bone marrow
CLP: Cecal ligation and puncture
LPS: Lipopolysaccharide
Ly6C^high^: Inflammatory monocytes
Ly6C^low^: Anti-inflammatory monocytes
MHC: Major histocompatibility complex
MSCs: Mesenchymal stem cells
PBS: Phosphate-buffered saline
Tregs: Regulatory T cells
WJ-MSCs: Mesenchymal stem cells derived from Wharton jelly
